# Bridging gaps: a qualitative inquiry on improving paediatric rheumatology care among healthcare workers in Kenya

**DOI:** 10.1186/s12969-023-00935-3

**Published:** 2023-12-13

**Authors:** Angela Migowa, Sasha Bernatsky, Anthony K. Ngugi, Helen E. Foster, Peterrock Muriuki, Roselyter M. Riang’a, Stanley Luchters

**Affiliations:** 1 Department of Paediatrics and Child Health, Aga Khan University, Medical College East Africa, Nairobi, Kenya; 2 International Centre for Reproductive Health, Department of Public Health and Primary Care, Ghent University, Ghent, Belgium; 3 Department of Medicine (Divisions of Rheumatology and Clinical Epidemiology), McGill University Health Centre (MUHC), Montréal, Canada; 4 Department of Population Health, Aga Khan University East Africa, Nairobi, Kenya; 5 Brain and Mind Institute, Aga Khan University, Nairobi, Kenya; 6 Centre for Global Health Equity, University of Michigan, Ann Arbor, MI USA; 7 Population and Health Institute, Newcastle University, Newcastle upon Tyne, UK; 8 African Population and Health Research Centre (APHRC), Nairobi, Kenya; 9 Centre for Sexual Health and HIV AIDS Research (CeSHHAR), Harare, Zimbabwe; 10 Liverpool School of Tropical Medicine (LSTM), Liverpool, UK

**Keywords:** Paediatric Rheumatology, Health care workers, Interventions, Kenya, Sub-Saharan Africa, Health Systems, Rheumatology Work Force, Global Health

## Abstract

**Background:**

Due to the paucity of paediatric rheumatologists in Kenya, it is paramount that we explore strategies to bridge clinical care gaps for paediatric rheumatology patients in order to promote early diagnosis, prompt referral, and optimal management.

**Purpose:**

To identify proposed interventions which can improve the ability of non-specialist healthcare workers to care for paediatric rheumatology patients across Kenya.

**Methods:**

We conducted 12 focus group discussions with clinical officers (community physician assistants), nurses, general practitioners and paediatricians across six regions in Kenya. Interviews were conducted, audio-recorded, transcribed verbatim, and analysed using MAXQDA 2022.2 software.

**Results:**

A total of 68 individuals participated in the study; 11 clinical officers, 12 nurses, 10 general practitioners, 27 paediatricians and eight other healthcare workers. Proposed patient interventions included patient education and psychosocial support. Community interventions were outreach awareness campaigns, mobilising financial support for patients’ care, mobilising patients to access diagnostic and therapeutic interventions. Healthcare worker interventions include diagnostic, management, and referral guidelines, as well as research and educational interventions related to symptom identification, therapeutic strategies, and effective patient communication skills. In addition, it was highlighted that healthcare systems should be bolstered to improve insurance coverage and access to integrated multi-disciplinary clinical care.

**Conclusions:**

Study participants were able to identify potential initiatives to improve paediatric rheumatology care in Kenya. Additional efforts are underway to design, implement and monitor the impact of some of these potential interventions,

**Supplementary Information:**

The online version contains supplementary material available at 10.1186/s12969-023-00935-3.

## Introduction

Paediatric rheumatic diseases such as Juvenile Idiopathic Arthritis (JIA) are associated with significant mortality, morbidity and reduced quality of life [1]. It is estimated that about 2 million of these children live in Africa [2–8]. However, given the paucity of paediatric rheumatologists (0.08 per million Africans versus 3–4 per million North Americans) and diagnostic resources, this could be an underestimate [9–14].

There have been various initiatives to help bridge the gap in the rheumatology workforce. This includes the UWEZO collaboration [15] between Kenyan, United Kingdom (UK) and Swedish rheumatologists that trained 500 healthcare workers across 11 sites in Kenya, and the EPAREP project (Enhancement of Paediatric and Adult Rheumatology Education and Practice) in Zambia [16]. Building the health workforce capable of offering clinical care to pediatric rheumatology patients and improving their performance for this noble task is a critical need globally [11]. As a result, it is important to get the view of the majority of the healthworkers treating pediatric rheumatology patients on how to improve clinical care offered.

The United Nations’ Children’s Fund (UNICEF) global ‘call to action’ (in 2021) emphasised the need to strengthen health systems through a focus on equitable access, integrated and community-based care [17]. Exploring healthcare workers’ understanding, attitudes and perceptions towards paediatric rheumatic diseases is key prior to offering solutions to improve pediatric rheumatology clinical are [18].

Interventions to improve healthworker performance should consider the contextual factors that would impact their local effectiveness for patients and families [19]. In order to implement these interventions effectively, policy makers need to understand and address the contextual factors which can contribute to differences in local effects [19]. Researchers therefore must recognise the importance of reporting how context may modify clinical service delivery [19].

Implementation of the “knowledge to action cycle” promoted by the Canadian Institute of Health Research highlights the importance of identifying challenges that are likely to impact on the effectiveness of an intervention, and to also consider the potential strategies of achieving change. These two fundamental principles not only inform the choice of intervention, but also facilitates the context to be modified, and the intervention “tailored”, as part of the implementation process [19]. Interventions to improve the performance of existing health workers have the potential to impact very positively on patient morbidity and mortality in an underserved sub-Saharan context.

In light of the above, we initially interviewed a cohort of healthcare workers across the Republic of Kenya to understand the challenges that they face in offering care to paediatric rheumatology patients in Kenya [17]. In the same focused group discussions, participants were interviewed to ascertain plausible solutions to help mitigate the challenges encountered. We thus aimed to identify interventions to improve the clinical care offered to paediatric rheumatology patients in the Republic of Kenya, as perceived by non-specialist healthcare workers.

## Methods

Our study incorporated the COnsolidated criteria for REporting Qualitative studies (COREQ Additional file 1: Appendix 1) as a reporting guide for qualitative research developed to promote explicit and comprehensive reporting of interviews and focus groups.

This was an exploratory qualitative study involving 12 focus group discussions conducted between September and November 2021 with 68 healthcare workers (HCWs) including clinical officers (physician assistants), nurses, general practitioners, and paediatricians. Participants were recruited from across the Kenyan Republic through the six regional branches of the Kenya Paediatric Association (KPA) namely Nairobi, North Rift, South Rift, Central, Coast and the Lake Region. In brief, participants were recruited through ‘snowball sampling’ and each cadre was then divided into 3 different Focus Group Discussions (FGDs) that were conducted virtually by AM (primary investigator, a paediatric rheumatologist) and PM (co-investigator qualitative researcher) through Zoom Communications (copyright 2021) using a standard interview guide that focussed on plausible interventions and implementation strategies. Data was recorded and transcribed verbatim. Notes of the focus group proceedings were used to cross-check for consistency. All forms, recordings and transcripts were managed according to ethical guidelines.

### Data analysis

A reflective thematic analysis was conducted using MAXQDA 2022.2 software [18]. Data familiarization was done by going through each quote by participants to deduce the key message. Initial coding where themes and sub-themes were grouped into categories was done by AM and RMR (co-investigator qualitative researcher). This followed a series of interactive meetings among members of the research team where linkages in themes were identified. Codes were merged into categories and themes. We completed a ‘member checking’ [20] process to check the accuracy of our findings.

### Ethical considerations

Ethical approval was obtained from the Aga Khan University, East Africa Institutional Research Ethics Committee (Ref 2021/IERC-50(v2)) and a research permit was obtained from the National Commission For Science, Technology & Innovation (NACOSTI/P/21/11789). Informed consent was obtained from all participants before data collection.

## Results

### Participants

Among the 68 participants, 78% (53/68) were female and the mean age was 36 years (inter-quartile range 31–40 years). Fifty percent of the respondents (34/68) worked in the public sector. Among those invited, one paediatrician and three general practitioners declined to participate due to lack of time and limited interaction with paediatric patients respectively. Table 1 below illustrates the biodemographic characteristics of the participants. Supplementary Fig. 1 shows the geographical distribution of study participants.

**Table 1:** Biodemographic characteristics of participants

Variable	Number *N* = 68	Percentage (%) *N* = 100%
**Gender**		
Female	53	78.00%
Male	15	22.00%
**Region**		
Nairobi Region	12	17.64%
Lake Region	16	23.50%
North Rift Region	7	10.29%
South Rift Region	12	17.64%
Coast Region	8	11.70%
Central Region	13	19.11%
**Healthcare Sector**		
Public sector	34	50.00%
Private sector	34	50.00%
**Cadre**		
Clinical Officers	11	16.17%
Nurses	12	17.64%
General Practitioners	10	14.70%
Paediatrician	27	39.70%
^a^Others	8	11.76%

^a^Research nurse, emergency physician, family physician, general physician, sleep coach, cardiac technologist, neonatologist, maternal-newborn specialist

Below are the interventions proposed by participants. These include patient-centered interventions on both an individual and community level, health worker interventions, and health systems interventions.

### Patient and caregiver psychosocial support and advocacy

#### Individual interventions

##### Patient education

One of the major challenges faced by healthcare workers regarding paediatric rheumatic patients is lack of understanding of the disease. Participants proposed that there should be a comprehensive education program for patients and their guardians to explain paediatric rheumatic diseases, their natural history, complications, therapeutic interventions and adverse effects. This will help guardians and parents improve how they manage and care for their children.

*“I think, the emphasis should be on the parents. Once parents understand this condition, this child is always being cared for well. But unless the parent understands and accepts that condition, it’s usually very difficult for these parents to care for this child.” *55 year old Female Nurse

##### Patient psychosocial support and advocacy

Participants proposed raising awareness about paediatric rheumatology and offering psychosocial support to families and patients as they battle with stress, stigma and confusion. The protracted duration of uncertainty and misdiagnosis creates doubt among guardians and hence they seek alternative medicine.

“And then sometimes, some beliefs like this patient has been bewitched especially given the chronicity of paediatric rheumatology, you might find that sometimes there is a conflict in terms of what you are going to do as a clinician versus what they want to go and try at home.” 39 year old Female Paediatrician

“On my side, before I forget is that the parents or the caregivers they present so late because when they were at home they were trying to use the herbal medicine or anything topical so by the time they are coming to the hospital, they come when it’s late. ……"how is it that a baby has rheumatoid arthritis at this age" because for them they understand it’s for the older age….” 30 year old Female Nurse

Key counselling components proposed for children, caregivers and families by the participants include: explaining the natural history of disease, complications, treatment options, toxicity and highlight the multi-disciplinary nature of clinical care. Counselling should be culturally acceptable, explained in a language they can understand, and context specific to suit the child.

“I think we should also involve patients in their care in that we inform them of their condition. Despite the fact that they are children, we should explain to them in a more understandable way in their context, what is expected, pain management, how to manage at home, how to identify symptoms early before they get worse.” 59 year old Female Nurse

“Then the stigma, when the children start getting injections, it interferes with their self-esteem. It interferes with their education. And you find these children, it’s like now they are, it’s like special children…… it has interfered with their life…… I've seen children suffer a lot, especially their self-esteem and their education. And it affects them more when they are adolescents.” 46 year old Female Clinical Officer

Paricipants also proposed psychosocial support and advocacy through group therapy.

*“Parents of children with these rheumatological illnesses, should also have a meeting together .. and share experiences and they capacity build each other. They may form something like an association where they have a forum where they can share this information and experiences.” *52 year old Female Nurse

## Community interventions

### Raising paediatric rheumatologyawareness

Participants highlighted a paucity of knowledge and awareness of paediatric rheumatology in the community.

*“You know like the way you have fever, joint pain and you say I think I need to get a malaria test, I think I need to see a doctor, so for rheumatological conditions, people in the community don't have any knowledge about it.” *35 year old Female Nurse

Participants proposed the need for community outreach initiatives to not only create awareness of the disease but also help find unidentified cases, refer promptly and follow up defaulters. This can be done through churches, community health volunteers (CHV) and local leaders through chief’s barazas (local community gatherings), schools and home visits. This awareness helps motivate community members to mobilize resources to help subsidise the costs of diagnostics and treatment. It was proposed school teachers should be empowered through educational approaches to help them identify and support children once diagnosed.

*“Where previously I was working, I was in a community where we used to serve patients who used to live in the slum area. And we used to have the advantage of using the area chief, the sub-chief. Now those are the local administrators. Then there was the community health volunteers who were useful in mobilizing parents to bring children to the clinic. And there is also now, the other important contributors would be the churches….”* 36 year old Male Clinical Officer

### Follow-up care

Participants stated continuity of care and follow up of patients is often suboptimal which worsens clinical outcomes.

*“I feel another challenge is follow up. Being that this is a rare condition, not much has been put in it. So, when we do the follow up, if this parent fails to bring this child to the clinic, usually you get most of the time nobody is bothered the way we do when we are dealing with sickle cell, HIV”* 55 year old Female Nurse

*“The challenge is patients get lost to follow up and after sometime they come back probably even sicker than they were and they need an admission.”* 24 year old Male Clinical Officer

It was recommended that a comprehensive follow up framework be established to help minimize loss to follow up among patients and regular communication between referring healthworkers to share feedback on clinical progress of patients.

*“I feel like I want to follow up with the patient while they are nurtured in the ward or just go through the patient records of the same and then maybe if I meet the rheumatologist, then we would have a chit-chat about the prognosis and what happened and what her thoughts are....”* 33 year old Male General Practitioner

### Patient financial support interventions

Respondents were concerned that lack of finances for diagnostics and treatment at the family level poses a great impediment to offering the appropriate clinical care to paediatric rheumatology patients. Financial constraint is partially accelerated by misdiagnosis and several trial and error tests and therapies by health providers.

“….most of these patients they will have gone through so many facilities that you find that they are already frustrated, they have used so many resources such that by the time they are getting to you, they are financialy drained, they don't even have money for the most basic tests or even a repeat test or something like that. So lack of resources is one of the major challenges that's there… “ 24 year old Male Clinical Officer

Some proposed solutions include government subsidies, public–private partnerships to source for funding, expanding existing insurance coverage, and establishing welfare funds. It was highlighted that facilitating quick diagnoses helps to reduce costs associated with mismanagement.

“*I would say if there is one thing I would want general reduction in the cost of management. Management here I mean right from investigation, treatment and probably the follow-up of these patients because where I practice I do work in a private facility so I've been able to see these tests being ordered and for sure as somebody has said before, they are damn expensive. ” *37 year old Male General Practitioner

*“The other thing would be insurance. …most of the medications are not covered by the insurance company especially, the NHIF (National Health Insurance Fund-Kenya’s public Health Insurance Scheme). I think it would be helpful if we have maybe individuals who would be able to kind of influence the decision makers under the NHIF insurance company to probably cover some of these medications to make it easier for the patient to afford care.” *27 year old Female General Practitioner

*“From where I'm working the place is pretty expensive so what I would recommend is if they have a welfare kitty, they offer it to rheumatology patients so that rheumatology patients will be more, and if the patients are more, we are able to learn more while the consultant is taking care of the rheumatology patients.” *29 year old Female Nurse

### Health worker interventions

#### Clinical interventions

##### Diagnostic interventions

One of the major challenges facing healthcare providers is accurate diagnosis of paediatric rheumatic diseases. They expressed their frustrations associated with misdiagnosis that increases medical costs to patients and leads to mistrust of the healthcare providers.

*“Someone has been treated for let me say just malaria, respiratory tract infection, sepsis yeah, when they come here, once you do all those investigations, then you find the diagnosis is too late. So it’s also important to make sure that this information reaches to the other centers, the facilities that will enable medical practitioners to make a concrete diagnosis and help manage these cases”.* 36 year old Male Clinical Officer

As a result, participants proposed various intervention strategies that can be adopted to improve diagnosis. These included simplified, easy to access guidelines aimed at facilitating early and accurate diagnosis whilst mitigating against mismanagement. These algorithms should cater to well-resourced facilities, resourced limited facilities and help them rule out mimics of rheumatic disease (e.g. infection, malignancy).

*“We need to arm people with the knowledge about these cases so that at least everyone would have an index of suspicion and make the right diagnosis with the meager resources that we have and also in whatever setting that we are seeing our patients.”* 37 year old Male General Practitioner

*“But again we also need to define which tests to order and what is priority and in terms of treatment put it also within the guideline so that we don’t over investigate or undertreat or over treat patients. I think some protocols and guidelines on management of these patients will be a welcome idea for us..”* 39 year old Male Paediatrician

Participants gave various proposals on the design and content of the diagnostic algorithm and protocols: it should be simple, concise, easy to access, updated regularly and evidence based.

*“Mine would be very simple. At least someone should take a proper history then if there is a work aid that would give you a scope that would tell that now you need to do lab work.”* 36 year old Male Clinical Officer

## Disease management interventions

Participants proposed having standardized management guidelines to help harmonize the treatment options offered to paediatric rheumatology patients to improve their confidence and efficiency.

*“Just having harmonized guidelines, the way we have like HIV guidelines or the diabetes guidelines or cardiovascular diseases guidelines, so I think it would help having a Kenyan guideline or protocol such that…, so from history and physical examination, the diagnosis, medications and follow up that these patients require.”* 27 year old Female General Practitioner

### Referral interventions

Lack of a clear referral pathway in the health system was also identified to be a major challenge.

As a result, participants requested for referral guidelines and prompt feedback to help expedite diagnosis and improve patient outcomes.

*“…to give the best care for patients, we should be able to have an appropriate referral protocol…...to be able to get a system where we could easily get that patient to a rheumatologist and be able to give the best care even if we don't really have all that we need.”* 27 year old Female General Practitioner

*“Feedback upon referral because I have a good idea, I've referred but really there's not much feedback so that I know, this is what I was dealing with and I'll be able to go read around it and be better equipped for the next time I encounter such patients.”* 41 year old Female Paediatrician

### Follow up interventions

Participants suggested patient follow up guidelines be availed to help minimize disease and iatrogenic complications. Structured follow ups are postulated to help in peer mentorship, networking, help build the clinician’s confidence and promote continuity of care.

“And then even for follow-up, those are some of the challenges, a patient you might see them once and then the next time you give them another clinic appointment, they don't show up ….and then patients hopping from one hospital to another. You know, they are seeking help so they'll hop from one hospital to another…… so we really need to figure out how do we improve so that we can also be able to retain these patients for follow-up.“ 46 year old Female Paediatrician

### Research interventions

Participants expressed interest to be updated with latest research and emerging knowledge on paediatric rheumatology diagnosis, treatment and care. They also highlighted the importance of data in making policy decisions.

*“The other thing is even after diagnosis in terms of treatment, rheumatology is one area that keeps growing and diversifying and there's new studies being done. A lot of new treatment modules are coming up, newer molecules especially like immune modulators, things that were not there when we were back in school but there are newer things that are coming out.”* 46 year old Female Paediatrician

*“Data has a voice. Because number one, they'll ask you, how many have you seen? And the problem we have is now we haven't had the cases one either because we have not been able to make a diagnosis of children with rheumatology. But…they'll ask about the data. "How many?" "Have there been like complications?" which will make me now influence. But without data, I may not have a voice.”* 55 year old Female Clinical Officer

#### Educational interventions

Participants proposed trainings to increase knowledge. Training can be delivered through different formats, such as;

- Fellowship training schemes
- Departmental meeting and local / regional events
- ‘Trainer of trainers’ to cascade knowledge
- Hybrid seminars to facilitate access remotely and also in person

*“Then, the second thing is, also long term, just have a fellowship for rheumatology within the country. I'm not sure whether we have any in the country so just empowering more healthcare professionals, like paediatricians or family medicine physicians to take up these training so that we have access to subspecialists.”* 27 year old Female General Practitioner

### Educational content

#### Symptom identification

Participants requested for an intervention to increase their knowledge on paediatric rheumatology symptoms and basic musculoskeletal examination skills in order to raise their index of suspicion when they encounter these patients. Furthermore, education among guardians, teachers and patients will help raise awareness and improve their healthcare seeking behavior.

*“I would say ….lack of knowledge is the reason things are the way they are so creating awareness among the staff just to understand more about...because the reason we are able to quickly handle respiratory diseases is because we know so much about them, we've handled them so they don't scare us at all. So, if the same awareness was created on rheumatological diseases that would be a first step.”* 29 year old Female General Practitioner

#### Disease management strategies

Participants highlighted they would like sessions on the protocols and guidelines of disease management which is believed shall minimize harmful alternative medical practices. Participants would like to know exactly which questions to ask when taking a paediatric rheumatology history.

*“It’s like even as you are asking us those questions, it’s an eye opener. So, I'd invite somebody with knowledge. To teach us more about rheumatology. The approach, the management and if possible give us a guideline. Just like we have a paediatric protocol such that we have no issues with pneumonias, bronchial asthma, all that. So, if we get somebody to educate us first and then to be given a guideline and maybe a mentor would help.”* 55 year old Female Clinical Officer

#### Communication skills

Participants were of the opinion that it will be important to develop effective and inter-cultural communication skills to explain the prognosis to patients and explain diseases in a patient friendly language. In addition, this skill will be key to raise awareness about paediatric rheumatic diseases with policy stakeholders.

*“Just to add on to what R11 said, I think we should also involve patients in their care..... despite the fact that they are paediatrics, we explain to them in a more understandable way in their context, what is expected, pain management, how to manage at home, how to identify symptoms early before they get worse and also involving their parents, the clients themselves and the paediatricians in initiatives towards informing the community about the condition and making people aware of it.”* 59 year old Female Nurse

### Education delivery strategies

#### CMEs (Continuing Medical Education)

Among the proposed mode of knowledge delivery was continuous medical education held in person or virtually given its potential to spur interest in paediatric rheumatology among healthcare workers and its vast reach. It was recommended that they can be stratified as per the level of care offered in the respective facilities.

*“The best way to create awareness in a facility and where I work is through CMEs because when you hold a CME in a public facility it is attended by everyone like my colleague the one who just spoke before me said. You hold a CME, it would be attended by the nurses, the lab people and all that. So there are different cadres that don’t know about paediatric rheumatology, they'll learn about it. So if you have scheduled topics maybe monthly or by every two weeks you cover different topics, then you will empower everyone to know about it from the COs (* clinical officer-community physician assistants*) who cover OPD (*outpatient department*) to the lab people…….”* 30 year old Female General Practitioner

#### Case based discussions

Another suggestion was to have case based discussions as this was felt to provide real time learning of context specific cases.

*“I would say learn, learn, learn, like read, read, read, maybe get someone who has experience like Dr. Angela there to just go through cases and be able to know how to write and rightly do things and how to manage patients or even sometimes to work under her and see how she manages patients so you can be able to improve that.”* 27 year old Female General Practitioner

#### Conferences

Conferences were also proposed as a platform of educational exchange to learn from multiple stakeholders.

*“ I've been practicing for four years, I have never been called for a rheumatology conference. In Kenya, how many times have you heard of an asthma conference or the malaria ones are every month in the public sector but rheumatology, it’s barely spoken about. I wish I had more knowledge on it.”* 30 year old Female General Practitioner

#### Online interactive applications and programs

Participants proposed having trustworthy online interactive applications and programs as these provide information “on demand” as they work that offer a “one stop shop” for all their needs in care of patients.

*“I would prefer UpToDate (*https://www.uptodate.com*). I would say I find it easy to read, not in terms of the volume of words but think the explanation and in terms of laying out the management for each condition, I think is more favorable for me and also as my colleague has said, like most of the data, or most of the information that is given in UpToDate are up to date.”* 27 year old Female General Practitioner

#### Virtual consults

Due to the limited number of paediatric rheumatologists and geographical barriers, participants proposed implementation of virtual consults.

*“One thing that really helped with the management of one of the patients we had was really a virtual access to a paediatric rheumatologist and we would do a case presentation and discuss focused on the patient management and keep giving updates on how the patient is doing and what is the next step because most of these conditions are treated for quite some time.”* 36 year old Female General Practitioner

#### Online courses

Participants proposed online training sessions and tutorials to allow virtual exchange of knowledge especially in instances where access poses a challenge.

*“Mine would be start ECHO (Extension for Community Healthcare Outcomes) teaching in the module so that people share. The ones who are already experts in it, they can share their knowledge with the others so that it becomes easier to diagnose the patient’s early enough and put patients on treatment. On the other side, other than ECHO, an online course where at the end of it, someone earns a certificate, those would help them actually acquire CPD (continuous professional development) points.…..So it can be renewable, maybe once you get the certificate, you can renew it maybe after one year or two years or three years. That would have a big impact.”* 36 year old Male Clinical Officer

#### Health systems interventions

Participants recommended it will be important to strengthen the health systems to improve access to care and patient outcomes. Recommendations proposed include:

#### Improving diagnosis

Participants proposed availing simple diagnostic tools at various health facilities to help with prompt diagnosis in the backdrop of understaffing, busy clinics and lack of time for detailed clinical assessment. These tools include for example diagnostic and management algorithms.

*“ And I think the best way, like she said, for the specialist to create sort of a simple tool which you can use even in the deepest of mashinani (remote place) to help you think these investigations you can do, how to prioritize your investigations since they are expensive. Something like that.”* 35 year old Female Paediatrician

#### Improving access to clinical care

Participants highlighted that improving access to clinical care is equally important in improving patient outcomes. It was proposed that this can be done by improving access to medications and reducing health provider-patient population ratio through strategies such as having more clinicians at the outpatient level and having social workers take histories and provide counseling. This can be further facilitated by making services affordable, reaching out to organizations willing to support paediatric rheumatology patients through donations as part of their corporate social responsibility. Participants also proposed paediatric rheumatologists be trained to provide holistic integrated multi-disciplinary care where all pertinent health workers eg rheumatologists, opthalmologists, phyiostherapists, occupational therapists are available to offer a “one stop” shop service for patients.

*“The other challenge is cost of treatment. There is a patient who wants to be given maybe (Methotrexate) or IVIG (Intravenous Immunoglobulin) and the insurance is not willing to commit to pay and the patient is forced to commit that they will pay, all of us know IVIG is pretty expensive so this delays treatment and the more we delay treatment, the more we get more organs involved in the condition. So maybe the government would intervene and this drug be made readily available at a better cost. I think that would also help.”* 35 year old Female Nurse

*“Number one, transport because they would only get the injection at Thika level 5 hospital (county regional hospital). Number two, in public facilities, you’d find there are stock outs of the medication so they’d have to go buy them in the pharmacies. If a diagnosis is made, the closest facility where they are, that is where they should be getting the injection from instead of a child wasting a whole day coming to get an injection then misses school. It would end up affecting their life in the long run.”* 36 year old Male Clinical Officer

*“Some organizations where I am, they usually chip in to cater for some of these investigations and to pay the bills for these children when they have been managed”* 36 year old Male Clinical Officer

#### Integrated clinical care

Participants proposed through adapting a multi-sectoral approach for example through engaging stakeholders involved in infrastructure development, medical supplies and healthcare education, hospitals can implement integrated multi-disciplinary clinical care so that patients have a one stop shop to have their clinical care given these conditions often present with multi-systemic manifestations.

*“Yes, it’s true. Like they say from the personnel, once you get the knowledge we have to involve different personnel from different departments because if I make the diagnosis without the help of the lab investigation, I wouldn’t be doing much and most of the time I’ll just refer the baby or the child to another facility where they can get those services. So if I’m able to involve the lab technician and also if those ones are not available I can be able to expedite that to either the superiors or the management to provide the services or even allow to create more awareness in the rest of the facility.”* 28 year old Female General Practitioner

Table 2 below summarizes our study findings.

**Table 2:** Summary of Study Findings

Theme	Sub-theme	Sub-sub theme
A. Patient and Caregiver Psychosocial support and Advocacy	I)Individual Interventions	1. Patient Education 2.Patient Psychosocial Support and Advocacy
	II)Community Interventions	1. Raising awareness 2. Follow-up Care 3. Patient Financial Support Interventions
B. Health worker Interventions	I)Clinical Interventions	1. Diagnostic Interventions 2. Disease Management Interventions 3. Referral Interventions 4. Follow-up Interventions 5. Research Interventions
	II) Educational Interventions	1) Educational Content i) Symptom Identification ii) Disease Management Strategies iii) Communication Skills
		2) Mode of Delivery i) Continuous Medical Education ii) Case Based Discussions iii) Conferences iv) Online Interactive Applications and Programs v) Virtual Consults vi) Online Courses vii) Online courses
C. Health System Interventions	1. Avail Diagnostic tools	
	2. Improve access to clinical care	
	3. Multi-sectoral approach to integrated multi-disciplinary clinical care	

**Fig. 1:**
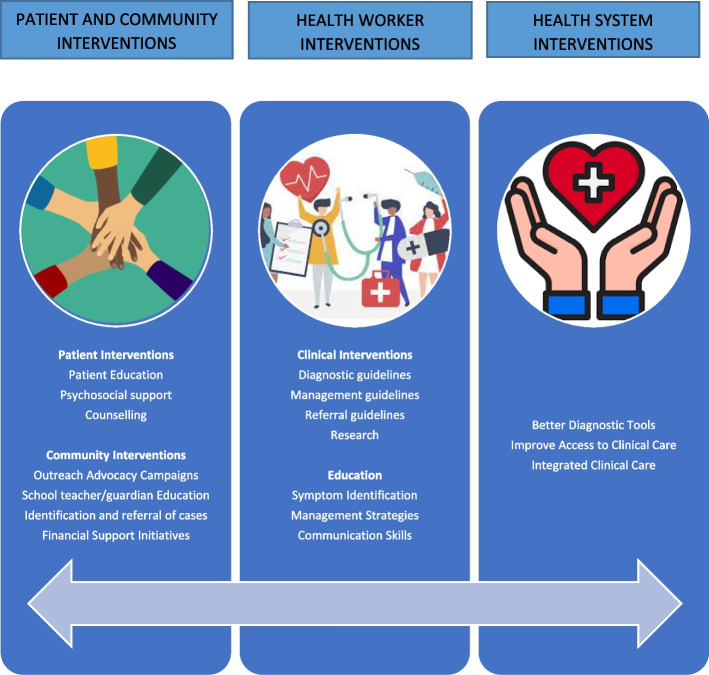
Conceptual Framework of Interventions to Improve Paediatric Rheumatology care in Kenya (Authors’ creation)

Figure 1 below captures the proposed interventions into a conceptual framework.

## Discussion

Our study identified interventions to promote patient and caregiver psychosocial support, healthworker interventions and health system interventions. Patient and caregiver interventions proposed are education, raising awareness in the community, follow up care and patient financial support interventions. Health worker interventions suggested included clinical interventions with guidelines and educational initiatives focused on symptom identification, management strategies and communication skills delivered through various virtual and in-person platforms. The health system interventions recommended were an integrated multi-disciplinary care model, through a multi-sectoral approach that involves the various stakeholders in health.

### Patient and caregiver psychosocial support and advocacy

Optimizing chronic disease care requires involvement of patients and their families as partners in the doctor–patient relationship [21]. Patient empowerment can be defined as having knowledge and being motivated to influence one’s health [22]. Our study participants emphasized it was necessary to empower patients by providing the necessary knowledge, skills, and understanding of their disease to allow effective self-management. Increasing patient empowerment can help promote efficient use of the health care system by reducing trial and error health visits and interventions [22].

Therapeutic patient education delivered by healthcare professionals can empower patients to engage in informed participation in clinical decision-making and effectively manage their conditions [21]. Several patient education interventions have been shown, in clinical trials, to improve medical outcomes, self-efficacy and satisfaction with treatment among patients with chronic conditions [21]. Nonetheless, patient education alone is often insufficient for generating and maintaining behaviour change, as health decisions are typically driven by multiple factors, not merely lack of knowledge [23]. It is important to combine psychological theory, research evidence, and patient/provider perspectives to promote appropriate health seeking behaviour and improve health outcomes [23].

Our study revealed that a key pillar in psychosocial support and advocacy was patient education and patient empowerment. Similarly, the majority of trial interventions to improve chronic disease outcomes in adults (reviewed recently [22]) include one or more of the following: self-monitoring, disease management training, exercise programs, and enhancing communication skills. These concepts were desirable outcomes of patient centered interventions proposed by our participants.

### Healthworker interventions

The quality of medical care varies across facilities [19]. Addressing the substantial variation in care quality produced by existing health workers due to their unique context may be a more feasible immediate-term solution [19]. Robust evidence about context specific interventions to improve health worker performance is key for success [19]. In a systematic review by Blacklock and colleagues about the impact of contextual factors on the effect of interventions to improve health worker performance in sub-Saharan Africa, they identified three staff-related themes namely: absolute shortages of staff, the inadequacy of existing knowledge and skills, and erosion of personal motivation to effect change [19]. Our participants reiterated the above themes by highlighting the paucity of health workers as evidenced by the long queues and wait times experienced by patients [17]. They further highlighted that lack of knowledge in paediatric rheumatology makes them lack confidence in management of these patients [17]. This demoralizes them and results in patients being ‘pushed on’ through the health system without getting any significant help [17]. This is exacerbated by high staff turnover, inadequate initial basic education, and lack of ongoing training [19].

Consequently, participants in our study proposed educational platforms, diagnostic aids, management, referral and follow up guidelines for the paediatric rheumatology patients they encounter. Similarly work by Blacklock and colleagues emphasized that training to impart knowledge to health workers should be accompanied by support and reinforcement infrastructure for meaningful change in clinical practice [19]. Participants proposed a variety of formats that are useful to improve access to education, diagnostic aids and management approaches. These included online platforms targeting a broad reach of healthcare workers, many of whom may have geographical, funding or time restraints. Many resources are already available; some are free (e.g. the PAFLAR webinar series [16] https://paflar.org/activities/ and the Paediatric Musculoskeletal Matters portfolio www.pmmonline.org which includes the pGALS basic MSK clinical examination https://www.pmmonline.org/doctor/clinical-assessment/examination/) while others are at a cost (e.g. UpToDate). The Project ECHO platform ( https://echo.unm.edu/) creates a community of learning and facilitates network development [24]. The challenge is to raise awareness about their existence and value to the target audiences.

Our study highlighted how expensive diagnostics and therapeutic interventions pose a challenge in achieving optimal clinical care in resource-poor settings. Alhassan et al. emphasized that patient and community factors such as poverty, while important, cannot be easily reformed, and highlighted the need to address other community issues such as language, cultural expectations, and transport issues [25]. Similarly, our study revealed the importance of engaging community leaders and stakeholders such as village elders and community health workers to promote health behavior change for sustainable impact. In addition, concurrent programs implemented by other agencies such as corporate entities and non-governmental organizations may have an important impact [25]. This was highlighted by participants citing examples of corporate organizations that offer financial assistance to some patients for diagnostic and therapeutic interventions.

It is important to create an ‘enabling’ environmental/organizational culture that supports evidence-based approaches such as collaborative learning, and the tailoring of approaches to suit the cultural, economic, political, and social context [26]. Our participants invoked these themes by suggesting frequent case based discussions and continuous medical education sessions as forums of knowledge exchange for paediatric rheumatology.

### Health system interventions

Progress in the management of communicable diseases and reproductive maternal and child health conditions, combined with demographic transition, have caused a shift in the burden of mortality and morbidity to noncommunicable diseases (NCDs) such as paediatric rheumatic diseases [27]. The long term management of NCDs requires integrated service delivery with the need for periodic reassessment and treatment modification [27]. This can be potentiated using digital technologies to reduce response time by using trained non-physician health workers, providing decision support, minimizing variability in the quality of delivered care, and optimizing monitoring and patient engagement, eventually reducing the cost of care and improving outcomes [27].

Our study participants also highlighted the above by proposing to have diagnostic and management algorithms available to improve their clinical decision making and ECHO sessions. In addition task shifting strategies were recommended to improve access to care by partnering with the social workers in the community and train them to take clinical history for paediatric rheumatology patients.

Innovations in service delivery should yield positive outcomes related to access, equity, quality, and responsiveness [27]. Well-designed, cost-effective studies are important to help policy makers use the limited health budgets to ensure maximum health benefits [27–30]. A key strategy is to include implementation research into the existing and proposed health initiatives to support generation of evidence for health system strengthening on strategically important outcomes [27–30].

Though our study has shed much light on possible ways to improve paediatric rheumatology care in Kenya, there remains much to learn about the specifics of interventions, e.g. in terms of optimal duration and/or frequency [22] and how best to measure factors like patient empowerment in health care evaluation [22]. Additional work also needs to be done to understand how community engagement and improved healthcare systems can ultimately reduce inequities [31]. Providing equitable access, of course, requires that healthcare systems have adequate infrastructure, governance, personnel, and other resources [32]. This includes strong partnerships, and good program management/co-ordination, to ensure success and sustainability [32]. A strong health system requires multi-sectoral engagement at many levels, including a community-based system for accessing local healthcare services [32].

Our study had several potential limitations. Since focus groups were conducted virtually, we were unable to study the full body language of respondents. In some instances, internet connectivity was a challenge and so some participants were not clearly audible; however, the majority of the time, the feedback was clear. Four potential participants were unable to join us due to a busy work load or other factors, but this is a problem in all focus-group studies.

## Conclusion

**Fig. 2:**
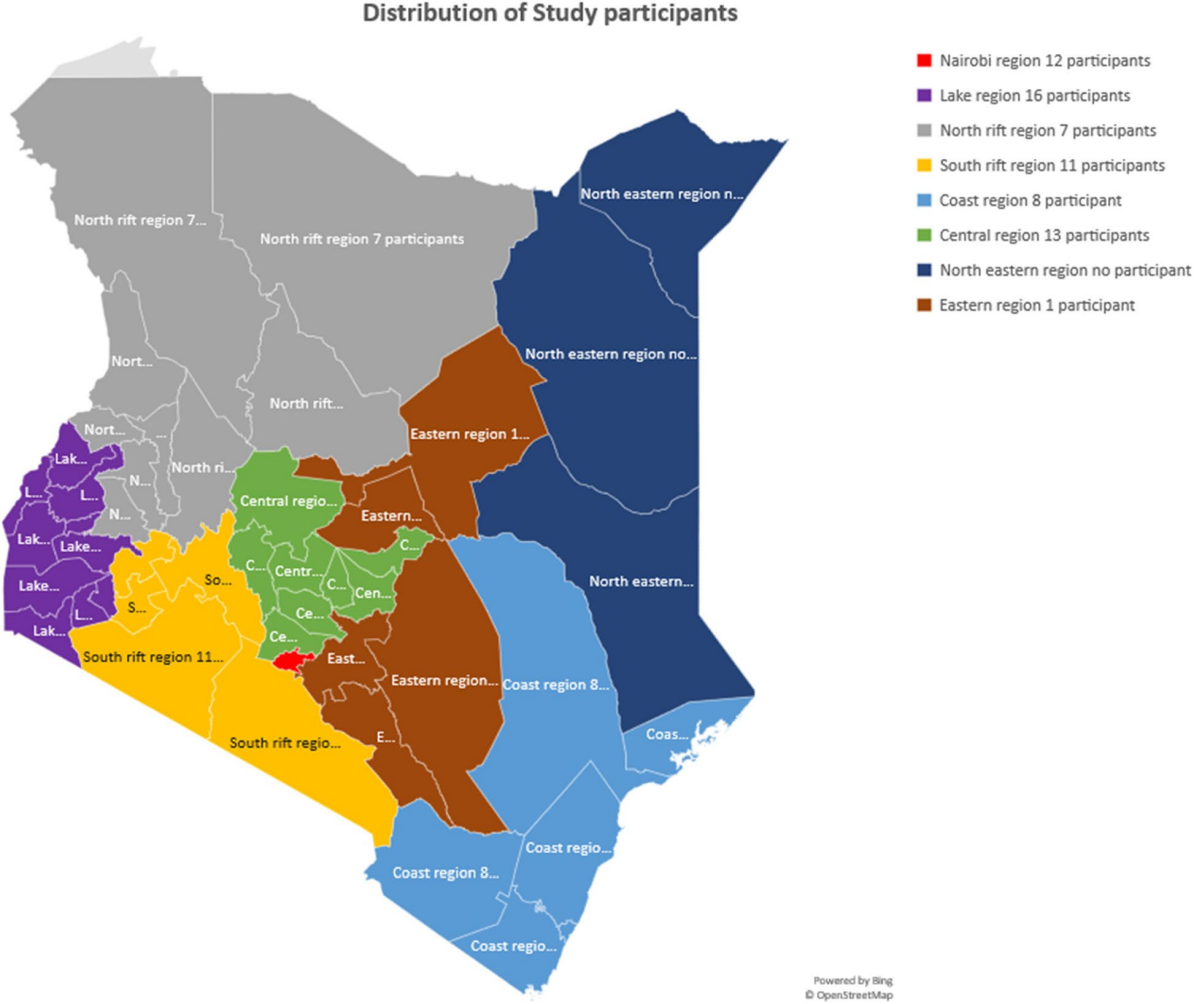
Geographical distribution of study participants

In summary, we were able to identify potential initiatives to improve paediatric rheumatology care in Kenya. Our work gathered opinion from ‘first line’ healthcare workers to generate potential solutions to improve care. Although the absolute number of participants was relatively small, we believe that it is representative of the target population of healthcare workers in Kenya given the geographical distribution of our sample (Table 1 and Fig. 2). Proposed interventions featured patient education and psychosocial support, community interventions (outreach/awareness campaigns and mobilising financial support for diagnostic/therapeutic interventions), and health worker interventions (i.e. guidelines, research, and provider education). In addition, it was highlighted that healthcare systems be bolstered to improve insurance coverage and access to medicines and integrated multi-disciplinary clinical care. Our findings pave the way to bridge care gaps in paediatric rheumatology services. Additional efforts are underway to design, implement and monitor the impact of some of these potential interventions, while engaging all stakeholders in the health ecosystem.

### Supplementary Information

**Additional file 1: Appendix 1.** Consolidated criteria for reporting qualitative studies (COREQ): 32-item checklist.

**Additional file 2: Figure 1.** Conceptual Framework of Interventions to Improving Paediatric Rheumatology care in Kenya (Authors’ creation).

## Data Availability

All de-identified data will be available on reasonable request to the corresponding author within a reasonable timeframe.

## Abbreviations

MAXQDA: Max Weber Qualitative Data Analysis
JIA: Juvenile Idiopathic Arthritis
EPAREP: Enhancement of Paediatric and Adult Rheumatology Education and Practice
UNICEF: United Nations Children’s Fund
COREQ: Consolidated Criteria for Reporting Qualitative Studies
HCWs: Healthcare Workers
KPA: Kenya Paediatric Association
FGDs: Focus Group Discussions
NACOSTI: National Commission for Science Technology and Innovation
CHV: Community Health Volunteers
NHIF: National Health Insurance Fund
ECHO: Extension for Community Healthcare Outcomes
CPD: Continuous Professional Development
IVIG: Intravenous Immunoglobulin
PAFLAR: Paediatric Society of the African League Against Rheumatism
PGALS: Paediatric Gait Arms Legs and Spine
NCDs: Non-Communicable Diseases
