# Supplementary material for: Bridging gaps: a qualitative inquiry on improving paediatric rheumatology care among healthcare workers in Kenya
Source: Pediatr Rheumatol Online J. 2023 Dec 13;21:144. doi: 10.1186/s12969-023-00935-3 (PMC10717234; doi:10.1186/s12969-023-00935-3)
Supplement: Supplementary file 2 — Additional file 2: Figure 1. Conceptual Framework of Interventions to Improving Paediatric Rheumatology care in Kenya (Authors’ creation). [file 12969_2023_935_MOESM2_ESM.docx]

Figures

Figure 1 below captures the proposed interventions into a conceptual framework.

HEALTH SYSTEM INTERVENTIONS

HEALTH WORKER INTERVENTIONS

PATIENT AND COMMUNITY INTERVENTIONS

**Figure 1: Conceptual Framework of Interventions to Improving Paediatric Rheumatology care** **in Kenya (Authors’ creation)**
